# Case report of refractory pericardial effusion associated with lymphatic fistula due to surgical injury during sternotomy

**DOI:** 10.1097/MD.0000000000009892

**Published:** 2018-03-02

**Authors:** Lijun Jiang, Tingting Tao, Junnan Zheng, Zhen Jia, Hongfei Xu, Yiming Ni

**Affiliations:** Department of Cardiothoracic Surgery, The First Affiliated Hospital of Zhejiang University, Hangzhou, China.

**Keywords:** pericardial effusion, lymphatic fistula, median sternotomy

## Abstract

**Rationale:**

A 35-year old Chinese female was admitted to hospital with refractory pericardial effusions 10 days post mitral valve replacement via median sternotomy. We performed an exploratory resternotomy and found lymphatic leakage on the surface of the diaphragm which was continuously emitting a light yellow fluid.

**Patient concerns:**

The patient complained of no obvious discomfort except for the concern of massive pericardial effusion drainage.

**Diagnoses:**

Exploratory resternotomy and biochemical testing lead to a supradiaphragmatic lymphatic fistula being diagnosed as the cause of the refractory pericardial effusion.

**Interventions:**

The fistula was closed with a continuous suture and no other fistulas were found after a thorough exploration.

**Outcomes:**

The patient was discharged home on postoperative day 5 and recovery was uneventful.

**Lessons:**

In this case a timely exploratory resternotomy proved effective in seeking the cause of and treating pericardial effusion following cardiac surgery.

## 1 Introduction

Pericardial effusion (PE) is a common complication of chronic heart failure, cardiac surgery, or as well as a variety of other diseases.^[1]^ The clinical manifestations and treatment of pericardial effusion following median sternotomy surgery have been widely reported. However, there exist limited reports confirming the actual causes of pericardial effusion.

Here we report a young female patient who presented with refractory pericardial effusion after mitral valve replacement. Exploratory resternotomy and biochemical testing lead to a supradiaphragmatic lymphatic fistula being diagnosed as the cause of the refractory pericardial effusion.

## 2 Case report

A 35-year old female with chronic hepatitis B infection and liver cirrhosis was referred to our hospital due to severe mitral valve regurgitation and atrial fibrillation. A decision was made to first treat the hepatitis infection and initiate liver protection therapy with a combination of Entecavir, the antiviral agent, polyene phosphatidyl choline and then operate on the heart defects. Once normal liver function was regained, she returned to our center for surgery. After all presurgery examinations had been carried out, we performed a mechanical mitral valve replacement and radiofrequency ablation. On day 5 postoperation the mediastinum and pericardium drainage tubes were removed. A routine echocardiogram found moderate fluid accumulation in the pericardium and fluid in the left pleural cavity. An arrow chest drainage tube was placed to treat the latter, however only 180 mL of a light yellow fluid was successfully drained. A computed tomography (CT) scan on day 9 showed a reduction in fluid in both the pericardium and the pleural cavity, so the patient was discharged on day 13.

At the time of discharge, an excessive amount of leachate (non-infection, non-bloody) was seen around the drainage wound, so the patient was instructed to attend her local hospital for further dressing and inspection of the wound. Ten days later, the patient went to the local hospital complaining of fatigue and chillness, physical examination revealed the wound had already self-healed and a CT examination showed moderate-to-severe pericardial effusion. A pericardial puncture was performed immediately and 500 mL of yellow liquid was drained. Approximately 1000 mL of yellow fluid was then drained daily for a total of 5 days. As there was no reduction in the volume of fluid being drained, the patient was readmitted to our hospital for further treatment. At admission, the patient complained of no obvious discomfort, her vital signs were stable, and no abnormal heart murmurs were found upon physical examination. The pericardial drainage tube place in the local hospital was still patent, and the characteristic of the drainage fluid remained liquid yellow. The volume of liquid drained daily remained at around 1000 mL.

**Figure 1:**
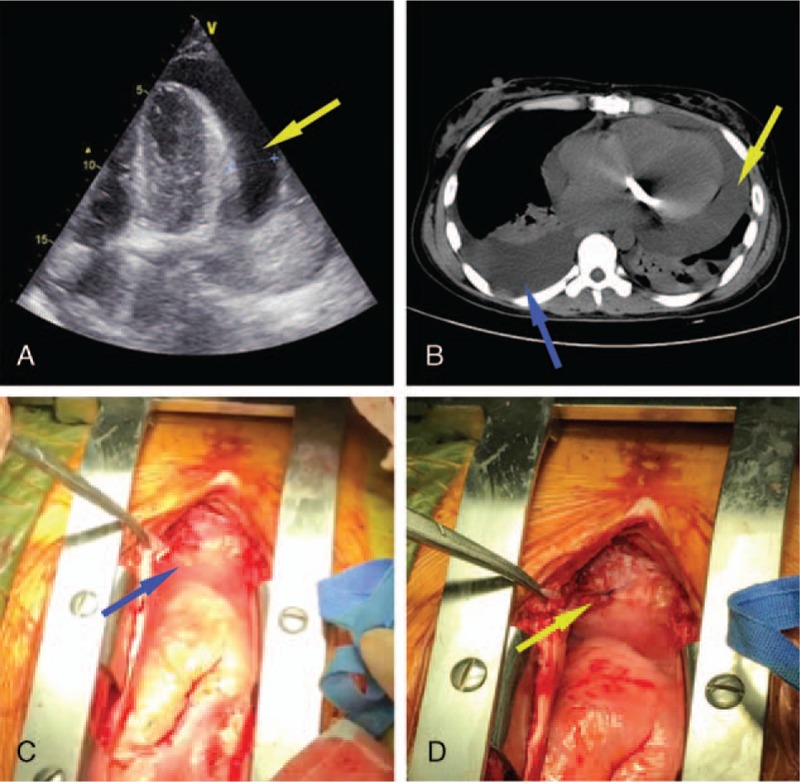
(A) Echocardiography showed moderate-to-massive pericardial effusion (yellow arrow). (B) Computed tomography showed massive pericardial effusion (yellow arrow), pleural diffusion on the right side and partial pulmonary atelectasis (blue arrow). (C) An opening of a duct, with light yellow fluid (blue arrow) flowing out continuously, on the diaphragm. (D) The visualized superior phrenic lymphatic fistula was closed with a continuous suture (yellow arrow).

On day 30 drainage liquid was sent for laboratory examination and was negative for chyle and no bacteria was found after a 10 day culture, and blood biochemical tests showed a total protein of 43.4 g/L, albumin 26.2 g/L, globulin 17.2 g/L, triglyceride 0.64 mmol/L, white blood cells 3.3×10^9^ /L and a C-reactive protein of 2.52 mg/L. Echocardiography revealed a pericardial effusion (Fig. 1A) and a normal mechanical valve at the mitral position with no obvious perivalvular leakage. On day 31, a CT scan showed a massive pericardial effusion, right sided pleural diffusion and partial pulmonary atelectasis (Fig. 1B).

The chyle analysis test was repeated on day 41, after the patient had been given food with a high fat content, and found to be positive. In the intervening period of the two chyle tests differential diagnoses for the pericardial effusion were ruled out, for example low osmotic pressure as a result of decreased liver function, low hydrostatic pressure due to decreased cardiac function post surgery, inflammation of the pericardium or an adverse drug reaction.

On the 43rd day postsurgery the decision was made to carry out an exploratory resternotomy during which an open lymphatic fistula with a continuous flow of yellow liquid was found immediately inferior to the position of the initial mediastinal drainage tube on the surface of the diaphragm (Fig. 1C and D). Thus a primary diagnosis of supradiaphragmatic lymphatic fistula was made. The fistula was closed with a continuous suture and no other fistulas were found after a thorough exploration. The sternum was not closed until a complete sternal hemostasis had been performed. The patient was extubated with 8 hours after surgery and discharged home on postoperative day 5. Recovery was uneventful.

## 3 Discussion

Causes of pericardial effusion include primary neoplasm, infection, cardiac surgery, idiopathic (viral), autoimmune, metabolic, drug-related and radiation therapy.^[2,3]^ However, the light yellow liquid seen in our patient was most likely lymph fluid associated with the supradiaphragmatic lymphatic fistula. Median sternotomy surgery can easily injure lymphoid tissue near the thymus.^[4–6]^ Lymphatic leakage on supradiaphragm is rare. Understanding the anatomical structure of the mediastinum is critical in avoiding surgical damage to the related tissues. The leaking fistula's proximity to the position of the initial mediastinal drainage tube suggests that the fluid was prohibited from leaving the lymphatic duct until the drainage tube was removed on day 5 post operation. Therefore, surgeons should be alert to sudden changes in the amount of fluid being effused, especially after drainage tubes have been taken out.

There are several ways to diagnose^[2]^ pericardial effusion including x-ray, CT, and electrocardiography; however, echocardiography has been proven to be most effective in the diagnosis of pericardial effusion. As for treatment, percutaneous drainage and pericardial window surgery are both practical ways to relief the symptoms for patients with malignant or noninfectious benign pericardial effusion, including pericardial tamponade.^[3]^ However, paper have shown that exploratory thoracotomy may be more effective in preventing refractory pericardial effusion.^[7]^

It cannot be overlooked that our patient's pericardial effusion and hydrothorax could have been on account of the underlying hepatitis B, liver cirrhosis, and hypoproteinemia.^[8]^ However, due to the character of hepatic hydrothorax and the manifestations of pleural effusions in different situations, as well as the unsatisfying result after an albumin infusion treatment, we finally judged that the light yellow drainage fluid is most likely lymph fluid caused by lymphoid tissues injury during the median sternotomy surgery. It was with all this in mind that we elected to proceed with a secondary exploratory resternotomy, the diagnosis of supradiaphragmatic lymph nodes fistula was reasonably proposed and the fistula was then closed. Six month follow-up has showed that the patient recovered uneventfully.

## 4 Conclusions

In summary, we reported a rare case of a visualized lymphatic fistula after cardiac surgery. Surgeons should, in addition to the thymus area, pay attention to the protection of the diaphragmatic surface and its surrounding lymphatic tissue during median sternotomy and pay particular attention any changes in the amount of fluid after drainage tubes are removed. Lymphatic fistulas caused by cardiac surgery can, on occasion, affect postoperative recovery. In case of refractory pericardial effusion after cardiac operation, a timely exploratory thoractomy can be effective in seeking the causes once other causes have been ruled out.
